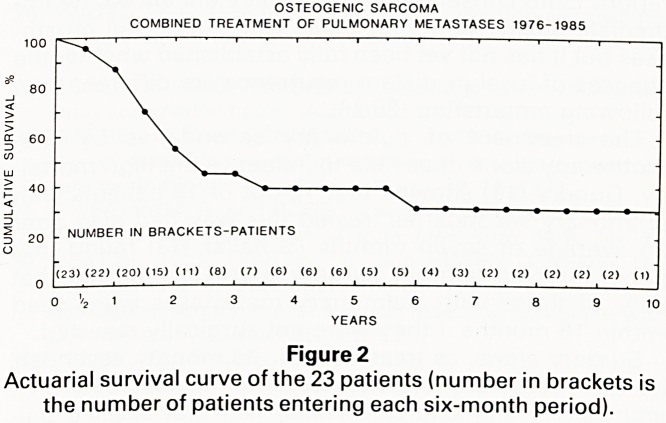# Management of Pulmonary Metastases in Osteogenic Sarcoma

**Published:** 1988

**Authors:** A. N. Al-Jilaihawi, J. Bullimore, M. Mott, J. D. Wisheart

**Affiliations:** Bristol Children's Hospital; Bristol Children's Hospital; Bristol Children's Hospital; Bristol Children's Hospital

## Abstract

Twenty-three patients presented with isolated pulmonary metastases from osteogenic sarcoma following primary treatment by amputation or limb salvage, combined with chemotherapy. The metastases were treated by conservative surgical excision, combined with chemotherapy; surgicl excision was repeated for recurrent pulmonary metastases provided there were none elsewhere.

Six patients are alive and disease free following their initial surgery. Of the remaining 17, 10 had recurrence confined to the lungs, and seven developed extra pulmonary metastases. The ten with isolated pulmonary metastases all had further thoracotomies but eventually seven died, as did all those with extra pulmonary metastases. There were in all 45 operations, with one hospital death and one serious complication. Actuarial survival at 1,3,5 and 7 years was 87, 45, 39 and 31% respectively. In the ten patients who had recurrence of isolated pulmonary metastases, survival at 1 and 3 years was 70 and 34%.


Bristol Medico-Chirurgical Journal Special Supplement 102 (1a) 1988

### Combined Chemotherapy and Surgery for Pulmonary Metastases from Osteogenic Sarcoma

**A. N. Al-Jilaihawi, J. Bullimore, M. Mott, J. D. Wisheart**
Bristol Children's Hospital

**ABSTRACT**

Twenty-three patients presented with isolated pulmonary metastases from osteogenic sarcoma following primary treatment by amputation or limb salvage, combined with chemotherapy. The metastases were treated by conservative surgical excision, combined with chemotherapy; surgicl excision was repeated for recurrent pulmonary metastases provided there were none elsewhere.

Six patients are alive and disease free following their initial surgery. Of the remaining 17, 10 had recurrence confined to the lungs, and seven developed extra pulmonary metastases. The ten with isolated pulmonary metastases all had further thoracotomies but eventually seven died, as did all those with extra pulmonary metastases. There were in all 45 operations, with one hospital death and one serious complication. Actuarial survival at 1,3,5 and 7 years was 87, 45, 39 and 31% respectively. In the ten patients who had recurrence of isolated pulmonary metastases, survival at 1 and 3 years was 70 and 34%.

### INTRODUCTION

Osteogenic sarcoma is a potentially lethal tumour. haematogenous spread carries the tumour first to the lung, and then elsewhere. In the pre-chemotherapy era treatment was by amputation and irradiation, but nevertheless 80% of patients had developed pulmonary secondaries within approximately two years (1–3). Untreated, 88% of these were dead two years later, and all by five years (2–4). Surgical excision of multiple, and if necessary, recurrent pulmonary metastases led to improved results with 28% surviving 5 years (5). The addition of chemotherapy to the surgical treatment was found to further improve survival (6), and since 1974 has been adopted by many centres as the treatment of choice for these patients (3,4,7–14). Since 1976 patients at this centre with pulmonary metastases have been treated with combined chemotherapy and surgical excision. The purpose of this paper is to report the results of this combined treatment with follow-up extending to a maximum of ten years.

### PATIENTS AND METHODS

Between 1976 and 1985 twenty-three patients prospectively entered this programme. There were 16 males and 7 females, aged between 8 and 27 years with a mean of 15.6 years. Amputation had been performed in 16 patients. Seven patients had had limb conservation using metal endoprostheses. All but one of the twenty-three patients had no evidence of pulmonary disease at initial presentation, and had received adjuvant chemotherapy as part of the primary treatment of the tumour; the development of lung metastases therefore indicated the failure of the adjuvant chemotherapy programme. The remaining patient already had pulmonary metastases when his primary tumour was diagnosed.

On diagnosis of pulmonary metastases, the patients were fully investigated to exclude metastases elsewhere, including either tomography or a CT scan of the chest and liver and bone scans.

### Chemotherapy

All patients received chemotherapy, the details of which were dictated by the previous failed programme of adjuvant chemotherapy. The most commonly used protocol was; Vincristine 1.5 mg/m$^2$ intravenously half an hour before an infusion of Methotrexate followed by folinic acid rescue. These drugs were given weekly for four weeks escalating the Methotrexate dose by 1 g/m$^2$ each week, to reach 4 g/m$^2$. The patient was then reassessed and surgery carried out 10 to 14 days from the last course of chemotherapy. Chemotherapy was restarted two weeks after surgery, the regimen being influenced by whether a favourable or unfavourable response to the pre-operative chemotherapy had occurred, as assessed by the degree of histological damage to the tumour. Doxorubicin was added to high-dose Methotrexate if the response was favourable, and other drugs including Actinomycin D, Bleomycin, Cyclophosphamide, cis-Platinum and Ifosfamide were used if the response was unfavourable.

### Surgery

Pulmonary metastases were usually multiple and in view of the possibility of further recurrence the principle of conserving lung tissue, with excision of the smallest possible amount with the tumour, was considered paramount (3–5,13). Small lesions tended to be peripheral and larger lesions were found deeper in the lungs; therefore simple enunleation was the preferred procedure for small lesions, while larger ones needed to be removed by more formal resections. For recurrence of metastases confined to the lung, further excision was performed, either alone or combined with further chemotherapy as appropriate.

Forty-five operations were performed, 29 for surgical removal of the initial metastases in twenty-three patients (six staged bilateral thoracotomies (13), and 16 for recurrent metastases (Table 1). In this series median sternotomy for bilateral metastases was not used (8,15). Wedge

Address for correspondence:
Mr J. D. Wisheart
Consultant Cardiothoracic Surgeon
Bristol Royal Infirmary
Marlborough Street
Bristol
BS2 8HW

**Table 1**

**Mortality and morbidity complicating surgery**

| | *Patients* | *Operations* | *Deaths* | *Complications* |
|---|---|---|---|---|
| Initial operations for pulmonary metastases | 23 | 29 | 0 | 1 |
| Operations for recurrence of isolated pulmonary metastases | 10 | 16 | 1 | 0 |
| TOTAL | | 45 | 1 | 1 |

Number of operations performed for initial or recurrent isolated pulmonary metastases showing the incidence of death or major complication.

resection was performed in two patients, segmental resection in three and lobectomy in one. Three patients had extension of their recurrent tumour to the chest wall or diaphragm, and in each of these the widest possible resection was undertaken; in all others, the metastases were removed by local excision.

**Follow-up**
Every patient was seen regularly in the outpatient clinic, examined for local recurrence or distant spread and investigated with at least a plain radiograph and/or a CT scan. While these patients are being followed indefinitely, this review was concluded on 31 December 1985.

**Statistical Methods**
Actuarial survival was calculated using the Kaplan Meier (16) method as described by Anderson et al (17).

### RESULTS

**Survival**

The results of the combined treatment are summarised in Figure 1; all patients survived the initial operations and six remain alive and disease-free. Metastases have recurred in 17 patients, in 7 of whom there was extra-pulmonary spread; all seven of these patients are dead. Ten patients had one or more recurrence of isolated pulmonary metastases and underwent a total of 16 further operations. In this group, there was one operative death and 6 subsequent deaths from tumour progression while 3 remain alive, 2 free of disease at 24 and 43 months from the time of the first metastatic recurrence and one with a further recurrence at 16 months. The three patients with chest wall extension of recurrent metastases have all died.

Thus there are 9 patients alive (range 13–126; median 60 months of whom one has disease at the time of reporting. Fourteen are dead (range 6–69; median 23 months), all of whom had progression of disease.

Actuarial survival for the whole group at 1, 3, 5 and 7 years is 87, 45, 39 and 31% respectively (Figure 2). For those who had one or more further operations for recurrence of isolated pulmonary metastases, survival at 1, 2 and 3 years is 70, 48 and 34% respectively. Eight of the 23 patients achieved a period of one year or more free of disease after the initial surgery, as did four of the 10 who had had further operations.

**Operative Mortality and Complications**
The only serious non-fatal complication was an episode of severe methotrexate toxicity. This arose following the first post-operative course of chemotherapy in a patient who had a small residual pleural effusion; this acted as a reservoir or 'third space' from which methotrexate re-entered the systemic circulation after the usual course of folinic acid rescue had been discontinued, resulting in prolonged exposure of susceptible tissues such as bone marrow, gut and mucous membranes to toxic levels of methotrexate.

**Figure 1**
Results of combined treatment.

**Figure 2**
Actuarial survival curve of the 23 patients (number in brackets is the number of patients entering each six-month period).

**Surgical Pathology**
One hundred and forty-five nodules were removed at the 45 operations with a mean of 3.4 (range 1–18). In all the patients a combination of viable and non-viable tumour cells was identified on histological examination.

**Possible Factors in Influencing Survival**
The interval between the presentation of the primary tumour and the metastases was considered: of 11 patients free of disease for less than one year, 3 were alive 11 to 110 months following excision of metastases. Among the 11 patients free of disease for more than one year, six are alive 11–109 months later. Examination of age and survival showed that seven of 13 children older than 15 years are alive but only two of 10 under that age.

However, these differences were not statistically significant, as was also the case for the other two preoperative or operative factors examined. However, a tumour-free interval of greater than 24 months after the excision of pulmonary metastases does indicate an improved prospect for survival. Seventeen patients were free of recurrence of disease for periods under 24 months and only 4 of them (23%) are alive at 30, 23, 11 and 11 months since operation; of 6 patients who were free of recurrence of metastases for over 24 months, 5 (83%) are alive at 9, 9, 6, 6, and 3 years since the operation while one patient died after 25 months ($P<0.05$). No other factors examined whether preoperative, operative or post-operative had a significant influence on outcome in this small series.

### DISCUSSION

The development of pulmonary metastases following limb amputation for osteogenic sarcoma was universally fatal in the past (2,13,19). Two changes have taken place in the primary treatment of the disease in recent years. First the introduction of chemotherapy in the early 1970s (7,20,21) and, more recently, the development of techniques of limb conservation (22,23). The use of effective chemotherapy has modified the behaviour of the tumour so that approximately 50% of patients achieve extended disease-free survival (21,24) and the frequency of occurrence of metastases within the first two years is greatly reduced (24). In a proportion of patients the development of metastases appears to be delayed, resulting in a longer tumour-free interval between the presentation of the primary and the appearance of metastases (21). Disease-free survival for two years after the primary treatment can no longer, therefore, be regarded as cure (21); dormant tumours may re-emerge after a longer interval (21), as happened in four of the 23 patients in this report. Limb conservation has greatly enhanced the immediate quality of life for those with successful prostheses but it has not yet been fully established whether the chances of local or distant recurrence are different than following amputation (25,26).

The treatment of pulmonary secondaries by chemotherapy alone does little to influence the high mortality. Gundry (11) showed that 12 out of 13 patients with pulmonary secondaries treated this way had died after an average of seven months; Schaller (13) found that none survived without surgery. Han (3) reported that 80% of those with pulmonary metastases were dead within 18 months if they were not surgically resected.

Surgery alone, as treatment for pulmonary secondaries has met with more success. After Torek (1930) (27), and Barney and Churchill (1939) (28) first excised pulmonary metastases in malignant disease, Sweetnam and Ross (29) reported that eight of 12 patients operated for solitary pulmonary metastases from bone tumours, had survived an average of 6.5 years. This led to an era of excision of the solitary secondary only, usually after some delay to confirm that no other metastasis was present (13,29). The use of surgery alone progressed so that conservative and, if necessary, repeated excision of multiple metastases was undertaken (3,5,9), and five-year survivals of just under 30% were achieved by this method.

By the mid 1970's the use of chemotherapy in the primary treatment of osteogenic sarcoma and a more aggressive surgical approach to pulmonary metastases were both sufficiently established in cancer centres to lead to the development of a multi-disciplinary approach to the management of pulmonary metastases using surgery, chemotherapy and, if needed, radiation (7,10,30). This yielded initially encouraging results with 65% survival at 18 months (10), 58% survival at three years (4) and similar early results from other workers (9). Longer follow-up, however, has shown that the 4–5 year survival is in the region of 40% in most studies (3,14); slightly higher figures have been reported by some workers (11,12), while a recent multi-centre European study found three year survival to be 20% (31). The philosophy of this multidisciplinary approach may be summarised as follows:

1. Osteogenic sarcoma is a systemic disease, which needs systemic treatment as well as local treatment (4).
2. The bulk of the gross disease may be surgically excised, while microscopic disease may be controlled by chemotherapy (24); adjuvant 'coned-down' irradiation may help to control local disease (8,24).

In practice this approach is applicable in patients with pulmonary metastases if the following criteria are met (5,24).

a) Control of primary disease.
b) No extrapulmonary metastases.
c) Pulmonary metastases are resectable.
d) The patient will tolerate the operation.

Our own experience over a ten-year period has involved the management of 23 patients by these principles. Survival at 5 and 7 years after the presentation of pulmonary metastases was 39% and 31% respectively. Six of the nine survivors have remained free of further recurrence and are living full and independent lives.

It has been claimed that various pre- and intraoperative factors will influence outcome, but there is no general agreement about this. However, post-operative events may also influence survival; in particular, recurrence of metastases would seem likely to have an adverse effect, though this has not been formally described or assessed in the literature. Excision of recurrent metastases by repeat thoracotomy is nevertheless widely advocated (3,5,8,13).

In this series recurrence of tumour appears to affect survival adversely. Those who recurred with extrapulmonary metastases all died. Of the ten patients who underwent repeat thoractomy for apparently isolated recurrent pulmonary metastases, three are alive but only two free of disease. Three year survival fell from 45% for the whole group after the first thoracotomy to 34% for this group following repeat thoracotomy.

In the past, two years of disease-free survival after the primary treatment was equated with cure (5); this is clearly no longer the case. However, two year disease-free survival after excision of metastases in this series correlated positively with prolonged survival and the risk of further recurrence would seem to be minimal.

For the majority of patients who died, an improved quality of life might still justify the rigours of combined treatment despite ultimate failure. Eight of the 23 patients achieved at least one year of freedom from disease after their first operation and four out of 10 did so after repeat thoracotomy. Although late survival after repeat thoracotomy appears to be reduced, the prospects of achieving one year of freedom from disease are still substantial. Therefore, repeat thoracotomy would appear to be justified in those circumstances.

### CONCLUSIONS

1. Combined surgical excision and chemotherapy prolongs the survival of patients with pulmonary metastases from osteogenic sarcoma.
2. The prospects for long-term survival are substantially improved after a two-year disease-free interval following resection of metastases.
3. The survival of patients treated for recurrent metastases confined to the lungs is less than that following initial pulmonary metastases.
4. The presence of extrapulmonary metastases, whether diagnosed pre-operatively or found at surgery, is associated with very short survival.
5. Among those who do not survive long-term, many nevertheless enjoy substantial periods of freedom from disease.

### REFERENCES

1. JEFREE, G. M., PRICE, C. H. G. and SISSONS, H. A. (1975) The metastatic problems of osteosarcoma. *Brit.J.Cancer* **32**, 87–107.
2. MARCOVE, R. C., MIKE, V., HAJEK, J. V. et al. (1970) Osteogenic sarcoma under the age of twenty-one. A review of 145 operated cases. J.Bone Joint Surg. **52-A**, 411–423.
3. HAN, M-T., TELEANDER, R. L., PAIROLLERO, P. C. et al. (1981) Aggressive thoracotomy for pulmonary metastatic osteogenic sarcoma in children and young adolescents. *J.Pediatr.Surg.* **16**, 928–933.
4. GIRITSKY, A. S., ETEUBANUS, E. and MARK, J. B. D. (1978) Pulmonary resection in children with metastatic osteogenic sarcoma. Improved survival with surgery, chemotherapy and irradiation. *J.Thorac.Cardiovasc.Surg.* **75**, 354–362.
5. SPANOS, P. K., PAYNE, W. S., IVINS, J. C. et al. (1976) Pulmonary resection for metastatic osteogenic sarcoma. *J.Bone Joint Surg.* **58-A**, 624–528.
6. BEATTIE, E. J., MARTINI, N., ROSEN, G. (1975) The management of pulmonary metastases in children with osteogenic sarcoma with surgical resection combined with chemotherapy. *Cancer* **35**, 618–621.
7. ROSEN, G., SUWANSIRIKUL, S., KWON, C. et al. (1974) High-dose methotrexate with citovorum rescue factor and adriamycin in childhood osteogenic sarcoma. *Cancer* **33**, 1151–1163.
8. HUANG, M. N., TAKITA, H., and DOUGLASS, H. O. (1978) Lung resection for metastatic osteogenic sarcoma. *J.Surg.Oncol.* **10**, 179–182.
9. ROSEN, G., HUVOS, A. G., MOSENDE, C. et al. (1978) Chemotherapy and thoracotomy for metastatic osteogenic sarcoma. A model for adjuvant chemotherapy and the rationale for the timing of thoracic surgery. *Cancer* **41**, 841–849.
10. JAFFE, N., TRAGGIS, D., CASSADY, J. R. et al. (1976) Multidisciplinary treatment for macrometastatic osteogenic sarcoma. *Brit.Med.J.* **2**, 1039–1041.
11. GUNDRY, S. R., CORAN, A. G., LEMMER, J. et al. (1984) The influence of tumour microfoci on securrence and survival following pulmonary resection of metastatic osteogenic sarcoma. *Ann.Thorac.Surg.* **38**, 473–478.
12. TELEANDER, R. L., PAIROLLERO, P. C., PRITCHARD, D. J. et al. (1978) Resection of pulmonary metastatic osteogenic sarcoma in children. *Surgery* **84**, 335–341.
13. SCHALLER, R. T., HAAS, J., SCHALLER, J. et al. (1982) Improved survival in children with osteosarcoma following resection of pulmonary metastases. *J.Pediatr.Surg.* **17**, 546–550.
14. FLYE, M. W., WOLTERING, G. and ROSENBERG, S. A. (1984) Aggressive pulmonary resection for metastatic osteogenic and soft-tissue sarcomas. *Ann.Thorac.Surg.* **37**, 123–127.
15. JOHNSTON, M. R. (1983) Median sternotomy for resection of pulmonary metastases. *J.Thorac.Cardiovasc.Surg.* **85**, 516–522.
16. KAPLAN, F. L. and MEIER, P. (1958) Non-parametric estimation from incomplete observations. *Am.J.Stat.Assoc.* **54**, 457–481.
17. ANDERSON, R. P., BONCHEK, L., GRUNKEIMEIR, G. L. et al. (1974) The analysis and presentation of surgical results by actuarial methods. *J.Surg.Res.* **16**, 224–230.
18. SWINSCOW, T. D. V. (1980) Statistics at Square One. 6th ed. London, British Medical Association.
19. DAHLIN, D. C., COVENTRY, M. B. (1967) Osteogenic sarcoma, a study of 600 cases. *J.Bone Joint Surg.* **49-A**, 100–110.
20. SUTOW, W. W., GEHA, F. A., VIETTI, T. J., et al. (1976) Multicrug chemotherapy in primary treatment of osteosarcoma. *J.Bone Joint Surg.* **58-A**, 629–633.
21. JAFFE, N., WATTS, H. G. (1976) Multidrug chemotherapy in primary treatment of osteosarcoma. *J.Bone Joint Surg.* **58-A**, 634–635.
22. ROSEN, G., MURPHY, M. L., HUXOS, A. G., et al. (1976) Chemotherapy, en-bloc resection, and prosthetic bone replacement in the treatment of osteogenic sarcoma. *Cancer* **37**, 1–11.
23. MARCOVE, R. C. and ROSEN, G. En-bloc resections for osteogenic sarcoma. *Cancer* **45**, 3040–3044.
24. WEICHSELBAUM, R. R., CASSADY, R. J., JAFFE, N. et al. (1977) Preliminary results of aggressive multimodality therapy for metastatic osteosarcoma. *Cancer* **40**, 78–1983.
25. SIMON, M. A., ASCHLIMAN, A. M., THOMAS, N. et al. (1986) Limb-salvage treatment versus amputation for osteosarcoma of the distal end of the femur. *J.Bone Joint Surg.* **68-A**, 1331–1337.
26. WATTS, H. G. (1980) Introduction to resection of musculoskeletal sarcomas. *Clin.Orthopaed.Rel.Res.* 1980; **153**, 31–38.
27. TOREK, F. (1930) Removal of metastatic carcinoma of the lung and mediastinum. *Arch.Surg.* **21**, 1416–1424.
28. BARNEY, J. D. and CHURCHILL, F. J. (1939) Adenocarcinoma of the kidney with metastasis to the lung cured by nephrectomy and lobectomy. *J.Urol.* **42**, 269–276.
29. SWEETNAM, R. and ROSS, J. K. (1967) Surgical treatment of pulmonary metastases. *J.Bone Joint Surg.* **49-B**, 74–79.
30. BEATTIE, F. J., MARTINI, N. and ROSEN G. (1975) The management of pulmonary metastases in children with osteogenic sarcoma, with surgical resection combined with chemotherapy. *Cancer* **35**, 618–621.
31. BERON, G., FULER, A., WINKLER, K. (1985) Pulmonary metastases from osteogenic sarcoma: complete resection and effective chemotherapy contributing to improved prognosis. *Eur.Paediatr.Haematol.Oncol.* **2**, 77–85.

## Figures and Tables

**Figure 1 f1:**
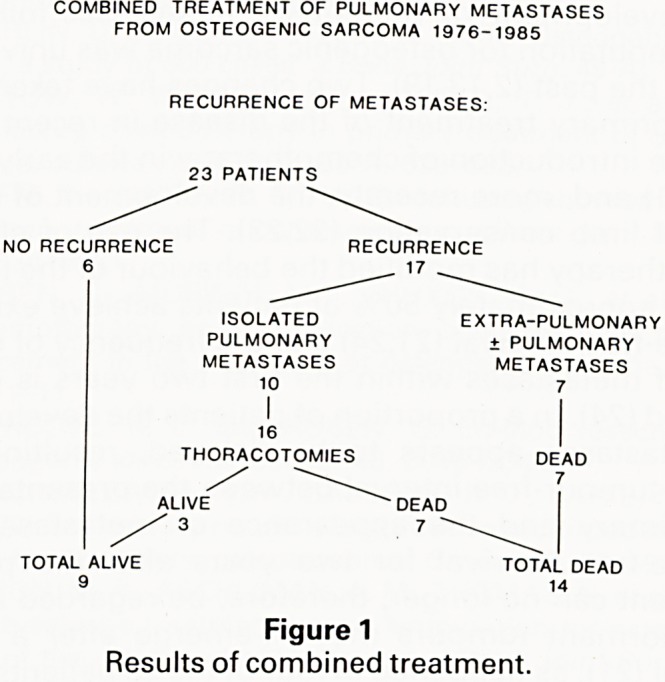


**Figure 2 f2:**